# A Rare Case of Primary Tubercular Ulcer Over the Posterior Oropharyngeal Wall

**DOI:** 10.7759/cureus.24868

**Published:** 2022-05-09

**Authors:** Farhat Q Khan, Prasad T Deshmukh

**Affiliations:** 1 Otolaryngology - Head and Neck Surgery, Jawaharlal Nehru Medical College, Wardha, IND

**Keywords:** extrapulmonary tuberculosis, rare tuberculosis involvement, posterior pharyngeal wall, oropharynx, mycobacterium tuberculosis

## Abstract

Tuberculosis (TB) is caused by *Mycobacterium tuberculosis*, an acid-fast bacillus that is predominantly transmitted through the respiratory system. Although TB most commonly infects the lungs, it may also affect other organs, resulting in secondary extrapulmonary TB. Extrapulmonary TB may occur alone or in conjunction with a primary pulmonary focus, such as disseminated TB resulting from self-inoculation with infected sputum, blood, or lymphatics. The lymph nodes are the most commonly seen extrapulmonary sites of TB. Oropharyngeal tubercular lesions are infrequent, and primary TB of the oropharynx is even more uncommon. Here, we present an unusual case of oropharyngeal TB in a young immunocompetent patient occurring without any evidence of pulmonary TB or cervical lymphadenopathy.

## Introduction

Tuberculosis (TB) continues to be a scourge in the Indian subcontinent. According to the latest World Health Organization statistics, an estimated 10 million people acquired active TB, leading to approximately 1.5 million fatalities, making it the leading cause of mortality after coronavirus disease 2019 [1].

TB is an infectious granulomatous condition caused by *Mycobacterium tuberculosis*, an acid-fast bacterium that is primarily transmitted through the respiratory system. Although TB commonly infects the lungs (90%), it may also manifest itself in other organs, resulting in secondary extrapulmonary TB [2]. Extrapulmonary TB may occur alone or in conjunction with a pulmonary focus, for example, in disseminated TB resulting from self-inoculation with infected sputum, blood, or lymphatics. The lymph nodes are the most common extrapulmonary sites of TB [3,4]. Oropharyngeal tubercular lesions are rare, occurring in 0.05-5% of all TB cases [1]. It is typically caused by direct inoculation of the mycobacterium present in coughed-up phlegm and is most often encountered in patients with sputum-positive pulmonary TB. Primary pharyngeal TB is a more uncommon condition [5].

Here, we report a rare and unusual case of oropharyngeal TB in a young immunocompetent male without any evidence of pulmonary cavitary disease or cervical lymphadenopathy.

## Case presentation

**Figure 1:**
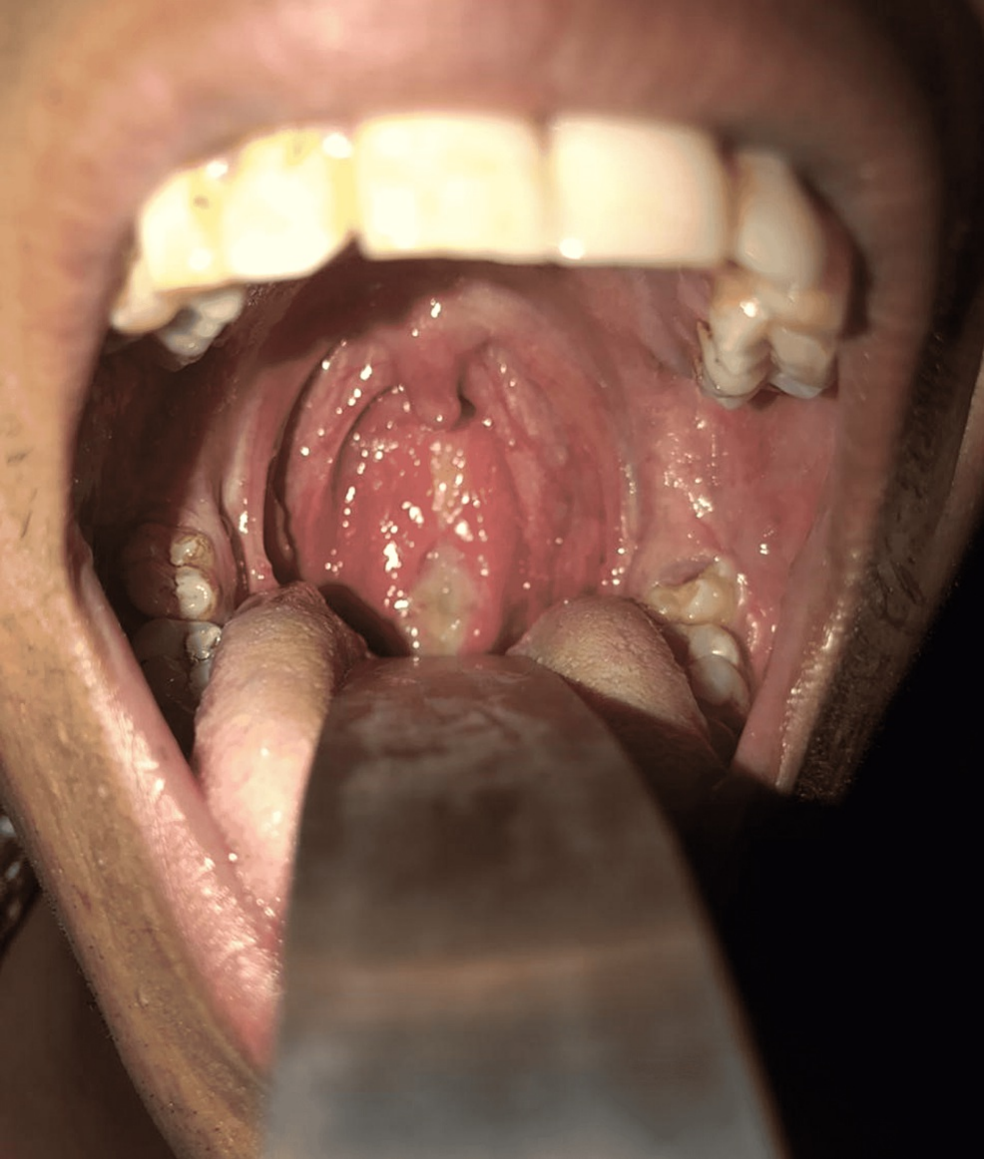
Ulcerative lesion seen over the posterior wall of the oropharynx.

A 20-year-old male presented to our ENT outpatient department with symptoms of discomfort and pain in the throat and difficulty in swallowing for six months. Additionally, the patient described an ulcer in the posterior pharyngeal wall that had been progressing slowly over the last six months. The patient also revealed a history of weight loss with a diminished appetite. There was no history of fever, cough, and hemoptysis. The patient was a non-smoker and non-alcoholic and no had no family history or direct contact with an active TB patient. He was fairly thin, and there were no palpable lymph nodes. Intraoral examination showed a single, oval, ulcerative lesion measuring 3 × 3 cm in the posterior wall of the oropharynx (Figure 1).

**Figure 2:**
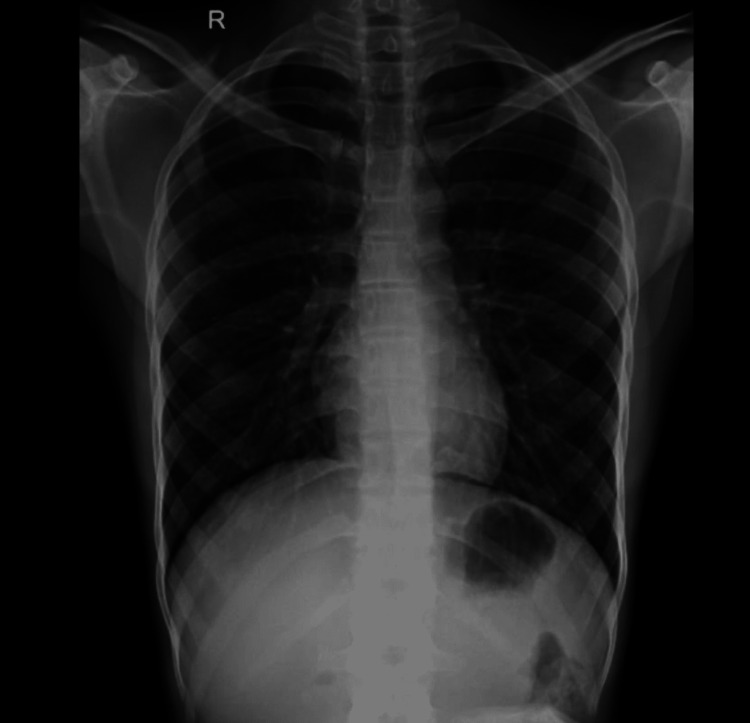
X-ray of the thorax (posteroanterior) view showing a clear chest.

The lesion was painful and did not bleed on probing. Routine examinations including hemogram, liver function test, and kidney function test were within normal range. Serological assays for Venereal Disease Research Laboratory test and human immunodeficiency virus were nonreactive. Acid-fast bacillus (AFB) and nucleic acid amplification tests detected no acid-fast bacilli in the sputum. An X-ray of the chest appeared normal (Figure 2).

**Figure 3:**
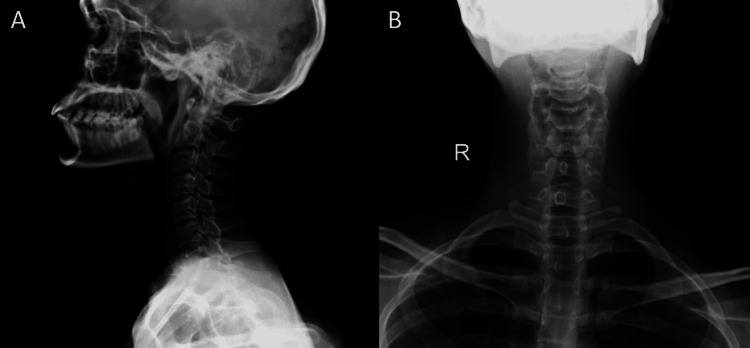
X-ray of the cervical spine lateral (A) and anteroposterior (B) view revealed no abnormality.

Radiological and orthopedic opinion was sought regarding the X-ray of the cervical spine that revealed no anomalies indicative of Pott’s spine (Figure 3).

Video-directed laryngoscope examination and diagnostic nasal endoscopy found no pathology. Contrast-enhanced magnetic resonance imaging of the neck revealed evidence of irregular growth in the left posterior wall of the oropharynx with thickening of the posterior-most part of the soft palate causing abrupt narrowing at the junction of the nasopharynx and oropharynx with no significant lymphadenopathy. A punch biopsy was carried out under local anesthesia, exhibiting features suggestive of caseous granulomatous lesion indicative of tuberculous etiology with no sign of malignancy. The patient was diagnosed with primary oropharyngeal tuberculosis and was immediately started on antitubercular regimen. Within a few months of initiation of treatment, there was complete resolution of symptoms and a significant reduction in the size of the ulcerative lesion. Moreover, the patient’s health status and overall performance improved.

## Discussion

TB is an infectious condition caused by *Mycobacterium tuberculosis*, also known as Koch’s bacillus. Transmission occurs when a person comes in contact with an active TB patient who coughs the bacilli in Pfluger’s droplets, causing primarily a pulmonary inoculation, or it may be transmitted to other parts of the body endogenously via the bloodstream or exogenously through infected sputum. Following endogenous spread, lymph nodes, bones, meninges, and the genitourinary system become involved [1,6]. Even at the peak period of 1930, the prevalence of TB affecting the nose, oral cavity, and pharynx was only 0.66% [5].

Tuberculous lesions of the oral cavity are extremely uncommon. The oropharyngeal tissue gets infected secondary to the expulsion of contaminated sputum, and according to Noone (1979), it may be associated with cervical lymphadenopathy [2,7]. In our case, there was no radiological indication of pulmonary TB, and sputum analysis was negative for AFB.

Primary pharyngeal TB is rare even in endemic areas and is reported as an exceptionally unusual manifestation of posterior pharyngeal wall TB. It manifests as an ulcer on the tonsil or oropharyngeal wall or as a nasopharyngeal granuloma [8]. Piasecka and Zeyland described that the bacteria are incapable of invading the healthy mucosa, and repeated swallowing action continuously clears the throat. Salivary enzymes, local antibodies, and oral saprophytes all aid in the resistance to such invasion [9]. The presence of trauma or inflammatory disease and unsatisfactory oral hygiene may offer a portal of entry to the bacilli. Cervical lymphadenopathy is often associated with the infection [10]. Hajioff et al. reported a case of primary TB of the posterior pharyngeal wall that presented with fever, throat irritation, and malaise [11]. Our patient exhibited a primary pharyngeal tubercular ulcer with dysphagia and odynophagia in the absence of fever or neck node which is a very rare presentation.

Often, on primary presentation, the tubercular oropharyngeal ulcer appears like a neoplasm [12]. Traditionally, when such ulcerative lesions do not respond to antibiotics, tissue biopsy is indicated to resolve the diagnostic conundrum [13]. The differential diagnosis for such unusual ulcerative lesions includes deep fungal infections, traumatic or reactive ulcers, infectious mononucleosis, Wegener’s granulomatosis, sarcoidosis, and malignancy such as squamous cell carcinoma [12].

## Conclusions

This case exemplifies the peculiar and unique manifestations of an extrapulmonary tubercular lesion of the posterior pharyngeal wall, demonstrating that *Mycobacteria *can infect practically any human organ. It is often challenging for physicians and ENT specialists to recognize bizarre presentations of TB. With the resurgence of TB globally, this deadly infection can no longer be disregarded.

Thus, any patient presenting with an ulcerative lesion of the oropharynx with an indolent course should be subjected to histological investigation to rule out malignancy, and clinicians should consider the possibility of TB. The patient must be started on an antitubercular regimen promptly upon confirmation of the diagnosis.
